# Antioxidant Activity of Milk and Dairy Products

**DOI:** 10.3390/ani12030245

**Published:** 2022-01-20

**Authors:** Magdalena Stobiecka, Jolanta Król, Aneta Brodziak

**Affiliations:** Department of Quality Assessment and Processing of Animal Products, Faculty of Animal Sciences and Bioeconomy, University of Life Sciences in Lublin, Akademicka 13, 20-950 Lublin, Poland; magdalena.stobiecka@student.up.edu.pl (M.S.); aneta.brodziak@up.lublin.pl (A.B.)

**Keywords:** milk, dairy products, bioactive compounds, bioactive peptides, total antioxidant capacity

## Abstract

**Simple Summary:**

Consumption of food products that are rich in natural antioxidants improves the antioxidant status of an organism through protection against oxidative stress and damage. Milk and dairy products (yogurt and cheese) accounting for approximately 25–30% of the average human diet are undoubtedly a rich source of compounds exhibiting antioxidant properties. The aim of the study was to present a review of literature data on the antioxidant potential of raw milk and dairy products (milk, fermented products, and cheese) and the possibility to modify its level at the milk production and processing stage. The antioxidant capacity of milk and dairy products is mainly related to the presence of sulfur amino acids, whey proteins (especially β-lactoglobulin), vitamins A, E, and C, or β-carotene. The processes of fermentation or cheese maturation are associated with the release of bioactive peptides, which are responsible for the level of the antioxidant status of the product. The use of probiotic strains significantly enhances the antioxidant status. The antioxidant status of milk and dairy products can be modified with the use of natural additives in animal nutrition or at the stage of milk processing. Herbal mixtures, seeds, fruits, and waste from the fruit and vegetable industry are used most commonly. It is worth emphasizing that regular consumption of natural dairy antioxidants minimizes the risk of development of civilization diseases (e.g., cardiovascular disease, cancer, or diabetes). It also slows down the aging process in the organism.

**Abstract:**

The aim of the study was to present a review of literature data on the antioxidant potential of raw milk and dairy products (milk, fermented products, and cheese) and the possibility to modify its level at the milk production and processing stage. Based on the available reports, it can be concluded that the consumption of products that are a rich source of bioactive components improves the antioxidant status of the organism and reduces the risk of development of many civilization diseases. Milk and dairy products are undoubtedly rich sources of antioxidant compounds. Various methods, in particular, ABTS, FRAP, and DPPH assays, are used for the measurement of the overall antioxidant activity of milk and dairy products. Research indicates differences in the total antioxidant capacity of milk between animal species, which result from the differences in the chemical compositions of their milk. The content of antioxidant components in milk and the antioxidant potential can be modified through animal nutrition (e.g., supplementation of animal diets with various natural additives (herbal mixtures, waste from fruit and vegetable processing)). The antioxidant potential of dairy products is associated with the quality of the raw material as well as the bacterial cultures and natural plant additives used. Antioxidant peptides released during milk fermentation increase the antioxidant capacity of dairy products, and the use of probiotic strains contributes its enhancement. Investigations have shown that the antioxidant activity of dairy products can be enhanced by the addition of plant raw materials or their extracts in the production process. Natural plant additives should therefore be widely used in animal nutrition or as functional additives to dairy products.

## 1. Introduction

Large amounts of oxygen free radicals are produced in the human organism through natural physiological processes and contact with the external environment as well as an inappropriate diet. It should be emphasized that not only exogenous but also endogenous sources of reactive molecules are very important because they lead to an increase in the number of molecules. As a result, there is a necessity of their reduction by an organism. In an aerobic organism, 5–10% of oxygen consumed with a high-fat and high-protein diet, contaminated food, and ultraviolet irradiation is converted into free radicals [1]. In conditions of normal metabolism, the generated free radicals are neutralized by the antioxidant system of the organism. Metabolic disorders lead to disturbances in the balance between free radicals and antioxidant reactions, which results in the accumulation of an excessive amount of free radicals in cells. Their excess in the organism associated with the imbalance between active oxygen species and antioxidant substances is referred to as oxidative stress. The excess of these molecules leads to substantial damage to proteins, lipids, and nucleic acids and, consequently, can be detrimental to the human organism. They lead to the development of tumors, neurodegenerative and neoplastic diseases, and disorders in the circulatory or nervous systems and accelerate degenerative processes [2,3,4,5,6]. As part of the defense against changes caused by reactive oxygen species, organisms have developed many mechanisms of prevention of excessive generation of these molecules and modification thereof into inactive derivatives. These mechanisms are based on both exogenous and endogenous compounds, which constitute a complex antioxidant system with nonenzymatic and enzymatic properties. The enzymatic antioxidant barrier consists of specialized enzymes (e.g., catalase (CAT), superoxide dismutase (SOD), glutathione peroxidase (GPx), and glutathione reductase) [7]. The second line of defense includes nonenzymatic antioxidants supplied to the organism with food [2,8,9]. Food antioxidants are compounds that “scavenge” free radicals through various mechanisms. They directly neutralize free radicals generated during the oxidation process, reduce peroxide concentrations, repair oxidized membranes, bind iron to reduce the production of reactive oxygen species, or neutralize ROS via the metabolism of short-chain fatty acids and free cholesterol esters [9,10]. High consumption of nutritional antioxidants protects against the risk of lifestyle diseases (e.g., cardiovascular diseases, cancer, diabetes, and obesity) [3]. It also contributes to an increase in the overall resistance of the organism to infections. An appropriate level of antioxidants in the organism is also important in the prevention of brain dysfunction. Many studies [4,6] have shown a lower incidence of such neurological diseases as cerebral ischemia, Parkinson’s disease, and Alzheimer’s disease in groups receiving antioxidant supplementation. The antioxidant capacity of milk and dairy products is the result of a complex balance between antioxidants and oxidants [11,12]. Oxidation processes exert a negative impact on milk quality (i.e., they shorten the shelf life and deteriorate the taste (appearance of an unpleasant aftertaste) and nutritional quality of milk) [13]. However, protein oxidation occurs independently of lipid oxidation. As shown by Havemose et al. [14], an elevated concentration of antioxidants is able to prolong the delayed phase of protein oxidation, thereby limiting the formation of dityrosine. Therefore, to improve milk properties, it is necessary to increase the level of bioactive ingredients with antioxidant properties. Antioxidant compounds play an important role in supporting and strengthening the defense mechanisms in the organism, which is useful for the prevention of some lifestyle diseases. Unfortunately, it has been proved that some synthetic antioxidants pose a potential threat in vivo [15]. Therefore, antioxidant compounds originating from natural sources are extremely valuable. One of the food sources of antioxidants is milk and dairy products. In regions and countries where milk consumption is high (North America, Australia, Europe, Argentina, and Pakistan) and amounts >150 kg/capita/year [16], milk and dairy products account for 25–30% of the average human diet [17]. These components are present in the protein (β-lactoglobulin (β-LG), lactoferrin (LF)), fat (vitamins E, A, β-carotene), and water (vitamin C, microelements: Sn, Zn, Fe, Mn) fractions [9,18,19,20,21].

The aim of the study was to present a review of literature data on the antioxidant potential of raw milk and dairy products (milk, fermented products, and cheese) and the possibility to modify its level at the milk production and processing stage.

## 2. Selected Nonenzymatic Antioxidants

The antioxidant potential of milk is directly associated with a content of components exhibiting antioxidant properties. Many authors emphasize that milk from dairy animals contains both enzymatic and nonenzymatic antioxidants, which are crucial in the prevention of the production of reactive oxygen species and help to strengthen the organism defense mechanism against oxidative stress [7].

### 2.1. Milk Proteins

Proteins are essential nutrients required for proper functioning of the human organism, providing all essential amino acids. Casein and whey proteins are the main proteins in milk. Casein accounts for approximately 80% of the total protein in cow milk. It is present mainly as micelles in macromolecular complexes. Whey proteins (i.e., α-lactalbumin (α-LA), β-LG, LF, immunoglobulins, serum albumin, and glycomacropeptides) constitute approximately 20% of milk proteins [22,23]. β-LG accounting for 50–55% is the main component of whey proteins [24,25]. It should be emphasized that human milk does not contain β-LG [26,27]. The antioxidant properties of proteins are primarily associated with their amino acid composition. Amino acids can act as antioxidants, mainly by limitation of the activity of their sulfhydryl groups (cysteine and methionine) or donation of aromatic residues (tryptophan, tyrosine, and phenylalanine). Moreover, the proper position of amino acids in protein sequences plays an important role in the antioxidant activity of proteins [28]. Whey proteins, in particular β-LG, are characterized by the highest antioxidant potential of all proteins from other food products. This is related to the high content of sulfur amino acids, especially cysteine, which is essential for glutathione synthesis [29,30]. In addition to antioxidant activity, β-LG hydrolysates have antihypertensive, antibacterial, and opioid properties [31,32]. LF has antioxidant activity as well. It was first identified in 1939 as a protein with high affinity for iron [33]. LF chelates iron, which increases its bioavailability and inhibits pro-oxidant effects. It suppresses the inflammatory response, increases the cytotoxicity of natural killer cells in vitro, and inhibits the release of ROS by leukocytes in inflammation sites [8,34]. However, the antioxidant capacity of LF decreases proportionally to saturation with iron [35]. LF stimulates the growth of the bacterial microflora by promoting the growth of selected probiotic strains. As suggested by Claeys et al. [36], LF can be completely inactivated only by thermal treatment of milk at 85 °C for 30 min.

As indicated by Kim et al. [37], in addition to the whey proteins, casein exerts an antioxidant effect as well. With their antioxidant properties, α-casein and β-LG can mitigate aging-related damage induced by oxidative stress through inhibition of cell aging and enhancement of differentiation and maturation of myoblasts. Similarly, the β-casein fraction exhibits high antioxidant activity due to the presence of proline residues. As shown by the comparison of the total antioxidant capacity of skimmed milk, casein, β-LG, α-LA, and various protein-milk mixtures reported by Cekic et al. [38], milk proteins (mainly β-LG and casein) are largely responsible for the total antioxidant capacity of milk. Many authors [26,39,40,41] indicated the effect of milk heat treatment on the antioxidant activity of dairy products, which is discussed in Section 5.

### 2.2. Bioactive Peptides with Antioxidant Properties

Proteins and their fractions are valuable sources of bioactive peptides exerting a positive effect on the functioning of the human organism [16,42]. Bioactive peptides have been classified as specific protein fragments with a positive effect on the organism [43]. The release of bioactive peptides from milk proteins in the gastrointestinal tract is a result of the action of such digestive enzymes as pepsin or pancreatic enzymes (trypsin, chymotrypsin, carboxy-, and aminopeptidases) [44,45] or milk processing with the use of starter cultures [46]. Currently, milk proteins are regarded as an important source of bioactive peptides, which are being increasingly identified in milk protein hydrolyzates and fermented dairy products [44,47,48]. Bioactive peptides are widely used due to their numerous health benefits (e.g., antioxidant activity). Their numerous antioxidant effects are the basis for the production of functional foods, nutraceuticals, and drugs of natural origin [49]. Milk and dairy products are a source of peptides with antioxidant properties, as shown in Table 1. These peptides have the ability to scavenge free radicals, chelate metal ions, and inhibit lipid peroxidation [5,8,22]. Numerous studies [1,5,22] have reported an interaction between the amino acid composition of peptides and antioxidant activity. Peptides usually consist of 5–11 amino acid residues, including hydrophobic ones (proline, histidine, tyrosine, tryptophan, or cysteine), whose free form also has antioxidant activity [50]. In experiments consisting in the treatment of hydrolysates of whey proteins (α-LA and β-LG) with enzymes (pepsin, trypsin, chymotrypsin, thermolysin, and Corolase PP), Hernandez-Ledesma et al. [51] identified 42 peptide fragments with the WYSLAMAASDI sequence exhibiting the highest antioxidant activity. Similarly, antioxidant activity is shown by β-casein and released peptides (e.g., those with the sequences VKEAMAPK, AVPYPQR, KVLPVEK, and VLPVPEK and αs1-casein (e.g., with the YFYPEL sequence)) [52]. Timón et al. [53] identified three peptides exerting a radical scavenging effect in Burgos cheese (i.e., peptides derived from αs1-casein (SDIPNPIGSENSEKTTMPLW) and β-casein (YQQPVLGPVRGPFPIIV and LLYQQPVLGPVRGPFPIIV). A number of antioxidant biopeptides have also been isolated and identified from β-LG hydrolyzed with the use of Corolase PP [54]. It has been shown that bioactive whey peptides, including the Ile-Pro-Ala tripeptide released from β-LG, can be used in the treatment of type 2 diabetes and obesity [55]. Bioactive peptides isolated from milk (VAGTWY) and Gouda cheese (LPQNIPP) can lower plasma glucose levels. Sommerer et al. [56] identified 28 small peptides with antioxidant activity from goat cheese, including 26 peptides from casein. Five new antioxidant oligopeptides from goat milk casein were identified by Li et al. [57]. In turn, Gupta et al. [58] identified two milk protein peptides with the sequences VKEAMAPK and HIQKEDVPSER from cheddar cheese fermented by Lactobacillus casei sp. casei 300. Peptides consisting of Met, Glu, Tyr, Lys, His, Cys, Val, and Pro have potent antioxidant activity [57,59]. As reported by Girgih et al. [60], the antioxidant properties of peptides can be enhanced by the presence of Trp, Tyr, and Pro. The researchers showed that the peptides WVYY (Trp-Val-Tyr-Tyr) and PSLPA (Pro-Ser-Leu-Pro-Ala) were the most active antioxidants characterized by 67% and 58% DPPH (2,2-diphenyl-1-picryl-hydrazyl-hydrate) scavenging capacity and 94% and 96% metal chelating activity, respectively. As suggested by Skrzypczak et al. [61], bioactive peptides (VLPVPQK (Val-Leu-Pro-Val-Pro-Gln-Lys) and QKAVPYPQRDMPI (Gln-Lys-Ala-Val-Pro-Tyr-Pro-Gln-Arg-Asp-Met -Pro-Ile)) isolated using Lactobacillus helveticus strains seem to have a high radical scavenging potential. The stability of antioxidant peptides in a simulated gastrointestinal tract was assessed as well [62,63]. Studies showed a slow increase in the rate of free hydroxyl radical scavenging in simulated gastric conditions. This may be attributed to the pepsin-induced decomposition of antioxidant peptides contained in fermented goat milk into smaller peptides with antioxidant properties. As reported by You et al. [63], greater numbers of peptide bonds were broken down in the process of digestion with pancreatin rather than pepsin. Investigations conducted by Woo et al. [64] confirmed that hydrolysis of milk proteins effectively increased their antioxidant activity ABTS test (2,2′-azino-bis 3-ethylbenzothiazoline-6-sulfonic), with the highest radical scavenging activity recorded in the process of casein digestion with trypsin.

### 2.3. Vitamins

Fat-soluble vitamins, mainly vitamin E, but especially α-tocopherol, as well as vitamin A and β-carotene, are the main antioxidants [93,94,95]. Their activity consists in organic free radical scavenging and inhibition of lipid peroxidation [96,97]. They also have the ability to quench singlet oxygen and hydroxyl radicals, effectively protecting DNA against oxidation [98]. Since they are present in fat globule envelopes, these vitamins prevent automatic oxidation of milk fat. Similarly, vitamin D3 is part of the nonenzymatic antioxidant system of milk, and 1,25-dihydroxycholecalciferol is its most active form. Its antioxidant effect consists in the inhibition of lipid peroxidation [99,100]. Vitamin D3 is mainly responsible for the regulation of calcium–phosphate metabolism and maintenance of calcium homeostasis in the organism [101,102]. An important antioxidant role in the organism is also played by vitamin C (ascorbic acid), which represents nonenzymatic water-soluble antioxidants.

The concentration of lipophilic vitamins in milk is directly related to animal nutrition. Higher levels of antioxidants (vitamin E, β-carotene, and retinol) were recorded in the milk of grazing cows compared with those fed concentrate- or silage-rich diets [103,104,105,106,107]. As shown by many authors [108,109], fresh pasture sward has a higher level of these vitamins than preserved fodder; therefore, grazing-based nutrition has a positive effect on their content in milk. Milk from grazing animals is also characterized by an increase in the content of vitamin D3 due to their exposure to UV [105,110,111,112,113]. Some investigations have shown a close relationship between the contents of β-LG and fat-soluble vitamins. As reported by Dolores-Perez and Calvo [114], the concentration of β-LG is positively correlated with the content of vitamin A, as this protein actively participates in the transport of small hydrophobic molecules (i.e., α-retinol). In turn, Bulgari et al. [115] demonstrated that the AA genotype of β-LG was associated (*p* ≤ 0.01) with a higher content of vitamin D3. Studies conducted by other authors [105,112] also indicate relationships between the contents of vitamin A and β-LG in milk. The amounts of vitamin C and lipophilic vitamins in milk decrease in mammary gland inflammation, as they are utilized during oxidation processes [116]. It should be emphasized that these compounds are sensitive to light and temperature, and greater vitamin loss is caused by UV radiation [21,117].

## 3. Methods for Assessment of the Antioxidant Activity of Milk and Dairy Products

Various methods are used for the determination of the antioxidant activity. They are based on the SET—single electron transfer: FRAP (ferric reducing antioxidant power), ABTS (2,2′-Azino-bis(3-ethylbenzothiazoline-6-sulfonic acid)), DPPH (2,2-diphenyl-1-picryl-hydrazyl-hydrate), and HAT—hydrogen atom transfer: ORAC (oxygen radical absorbance capacity) and TRAP (total radical-trapping antioxidant parameter) mechanisms. In the SET methods, the reaction mixture is composed of an antioxidant and an oxidant; the latter changes color in the reduction reaction (i.e., an electron transfer from the antioxidant to the oxidant). The results obtained with this method are often converted into Trolox equivalents (TEAC—Trolox equivalent antioxidant capacity). Methods based on the mechanism of the hydrogen atom transfer (HAT) reaction are recommended for the measurement of the deactivation of free radicals resulting from the donation of a hydrogen atom by the antioxidant. The antioxidant present in the sample and the model antioxidant with a known concentration, referred to as the “molecular probe”, compete with each other for the reaction with the peroxide radical. Each of these methods has a specific mechanism of action (Table 2). The methods are based on the determination of the effect of antioxidants on the rate of oxidation processes taking place in the sample (ORAC and TRAP), reduction of metal ions (e.g., iron (FRAP) or copper CUPRAC (cupric reducing antioxidant capacity)), and the ability to scavenge synthetic radicals (ABTS, DPPH) or measurements of the amount of lipid oxidation products or LDL fractions [118,119]. Three methods are most often used to assess the antioxidant activity of milk and dairy products (i.e., DPPH, ABTS, and FRAP) [120]. The ABTS method is based on 2,2′-azino-bis(3-ethylbenzothiazoline-6-sulfonic) cation radical. It is a spectrophotometric method for the determination of the ability of antioxidants to neutralize the blue cation radical generated from ABTS under the influence of sodium persulfate, which is manifested by a decrease in the solution absorbance. The method is widely used due to its simplicity, speed, and sensitivity [121,122]. DPPH is another compound used in the measurement of the reducing ability of antioxidants towards this reagent. This assay measures the loss of DPPH color (deep purple) at 515 nm after reaction with the antioxidant. The percentage of the remaining DPPH is calculated. The DPPH radical scavenging ability is expressed in other units in addition to% inhibition [122,123]. FRAP (ferric reducing antioxidant power) measures the reduction of ferric 2,4,6-tripyridyl-S-triazine (TPTZ) to a colored solution (blue) assessed at 595 nm using a spectrophotometer. FRAP measures reducing power but cannot detect compounds that act via radical quenching (H transfer), particularly thiols and proteins [123]. However, it should be noted that, although there are a number of methods for the assessment of antioxidant properties, their results are not standardized. Unfortunately, there are often discrepancies in results obtained from the same material analyzed using different methods and even in the case of the same material analyzed with the same method in different research laboratories [120,124].

## 4. Antioxidant Potential of Raw Milk, Effect of Animal Species, Diet, and Lactation Phase

The antioxidant potential of milk is determined by animal species, diet, and lactation phase.

As shown in some investigations [17,30,125,126], the species of the animal has a significant impact on the content of antioxidant components in milk. Compared with cow milk, which is the main material in the world production, sheep, camel, and buffalo milks contain higher levels of these ingredients, mainly β-LG, LF, vitamins A and E, and polyunsaturated acids. Interspecies differentiation in the total antioxidant capacity of milk determined using various methods has been shown (Table 3). Regardless of the method of determination, raw cow milk has the lowest antioxidant potential. In the assessment of the total antioxidant potential of raw cow and sheep milks using three methods (DPPH, ABTS, FRAP), Yilmaz-Ersan et al. [30] reported a lower antioxidant capacity of raw cow milk, which can be explained by the differences in their chemical compositions. Furthermore, Khan et al. [126] indicated statistically significantly higher values of antioxidant status (expressed as DPPH%) for buffalo milk as compared with cow milk.

The content of antioxidant components in milk and the value of its antioxidant potential can be modified via animal nutrition (e.g., the use of various natural additives to animal diet). Feed additives used in animal nutrition serve protective functions and act as regulators of metabolism. Consequently, they contribute to an increase in the immunity of animals exposed to stress (weaning, changes in diet, or transport), lead to efficient absorption of essential nutrients, and enhance the antioxidant protection provided by milk [132]. Examples of antioxidant activity of milk after supplementation of feed with natural plant additives are shown in Table 4. Grazing significantly increases the content of antioxidant components in milk, thus increasing its antioxidant potential [93,98,105,133]. The improvement is more difficult to achieve when cows are fed preserved fodder, especially silage [134,135,136]. A higher proportion of maize silage in feed rations for cows is one of the main factors of the lower content of vitamins and antioxidants in milk. Alves et al. [135] demonstrated that maize silage, which is most often used in cow nutrition, has low contents of carotenoids. Pumpkin silage is regarded as a valuable source of bioactive compounds, especially carotenoids and flavonoids [137]. As demonstrated by Halik et al. [138], the addition of pumpkin silage to diet for dairy cows significantly improved the nutritional value of colostrum, including the content of carotenoids. The colostrum antioxidant status was significantly higher as well. Similar results were obtained from studies of milk from cows fed carotenoid-rich diets [139]. Santos et al. [140] reported higher antioxidant activity of milk from cows receiving grape pomace silage in their diet. In turn, the supplementation of cow diet with synthetic and natural β-carotene had no effect on its content in milk and TAS value. However, the natural β-carotene exhibited higher availability than the synthetic compound [141,142]. Delgado-Pertíñez et al. [143] used dried orange pulp (DOP) (i.e., a waste from orange juice production) as an alternative component of ruminant diet in goat nutrition. It was shown that the addition of DOP to the feed ration significantly improved the health-enhancing value of milk and increased the level of vitamin E, phenolic compounds, and milk antioxidant capacity (ABTS). Grape pulp (GP) (i.e., the main by-product in wine production and a rich source of bioactive compounds) was used in animal nutrition as well. The addition of GP naturally enriched the feed with polyphenols and dietary fiber. An increase in the concentration of antioxidant bioactive compounds in milk was mainly observed. In turn, Chedea et al. [144] and Ianni et al. [145] reported no effect of a diet supplemented with GP on the health condition of cows and the chemical composition of milk. Herbs and spices are used as natural feed additives in animal nutrition. With their high content of biologically active substances (flavonoids, saponins, carotenoids, plant sterols, glucosinolates, or essential oils), they exert a positive effect on the animal organism, dairy production, and milk quality [146,147]. Importantly, spices and herbs are a natural source of antioxidants [120]. Most antioxidants contained in spices and herbs react with free radicals generated in the initial stage of autoxidation. Due to their antioxidant properties, rosemary, thyme, anise, buckwheat, black pepper, cinnamon, garlic, fenugreek, savory, and mint are used in animal nutrition most frequently [147,148,149,150]. Supplementation with 2% of a herbal mixture of yarrow, chamomile, nettle, turnip rape, plantain, and lady’s mantle had a positive effect on the health of the mammary gland in cows and on the nutritional value of their milk [151]. It has been shown that the fat content in milk has an impact on its antioxidant potential. Fat-rich raw milk has a higher antioxidant value than reduced-fat milk due to the lower content of lipophilic antioxidants in fat-soluble vitamins [12,152]. Chen et al. [153] reported similar findings. They determined the total antioxidant capacity of milk using the ABTS test and showed that cow milk containing 3% of fat exhibited a substantially higher antioxidant capacity than milk with lower fat content (0.5–1.5%) or skimmed milk. Additionally, the authors revealed positive correlations between the fat content and the antioxidant capacity of milk. As reported by Puppel et al. [154,155], the antioxidant capacity of milk may be related to both the dietary supplementation and the age of cows. The modification of diets for multiparous and primiparous cows with fish oil and linseed significantly influenced the antioxidant properties of their milk. In both groups, the supplementation contributed to an increase in the total antioxidant status, with higher values recorded in the milk from the primiparous cows. Other studies conducted by these authors [133] demonstrated that supplementation of the basic diet with maize grain improved the antioxidant capacity and antioxidant protection of milk by increasing the content of vitamin E in milk. It was also shown that addition of cow milk to green and black tea (50 mL of tea mixed with 50 mL of milk) resulted in an approximately 2.1-fold increase in their antioxidant potential [156].

The various physiological processes taking place during lactation are associated with the generation of reactive oxygen species. The animal organism utilizes antioxidants to reduce free radicals [9]. Mann et al. [71] underlined the effect of the lactation phase on changes in the antioxidant status of milk. The antioxidant capacity of milk from different breeds of cows (Sahiwal, Karan Fries, Holstein Friesian) was determined as the ability to reduce iron ions (FRAP test) and the free radical scavenging activity (DPPH test). Higher values were noted in colostrum and milk in the early stage of lactation (5–15 days). With time, the values of the antioxidant potential declined significantly. The authors suggest that higher levels of antioxidants in colostrum may be critical for the protection of the health of neonatal animals against oxidative stress. Annie et al. [157] measured the total antioxidant capacity (FRAP method) of milk produced by Vechur cows (native Kerala breed) and Malabari goats in different stages of lactation. It was shown that the antioxidant potential of milk changed significantly (*p* < 0.01) along the lactation period, with the highest potential noted in the early stage of lactation (5–15 days) compared with mid (90–120 days) and late (>150 days) lactation. As shown by Kapusta et al. [158,159], there is a clear relationship between the lactation phase and the level of enzymatic and nonenzymatic antioxidants in milk in high-yielding PHF cows. The highest total antioxidant status (TAS) in milk was demonstrated on the first days of lactation (≥8), but its level was found to decrease gradually on the subsequent days.

**Table 4:** Antioxidant activity of milk after supplementation of feed with natural plant additives (own work based on: [140,160,161,162,163,164,165,166,167,168,169,170]).

Animal	Additives to the Diet	Method	Antioxidant Activity	References
Cow	Control diet (without black rice and purple corn extracted residue)	DPPH	6.96%	[160]
	2% black rice and purple corn extracted residue		7.68%	
	4% black rice and purple corn extracted residue		9.76%	
	6% black rice and purple corn extracted residue		9.27%	
	Grape pomace extract	Folin–Ciocalteu	16.07 GAE mg/g	[161]
	Raw mulberry cultivars (*Yuesang 11*)	DPPH	146.04 mg of TE/g of DM	[162]
		ABTS	21.85 mg of TE/g of DM	
		FRAP	52.71 mg of TE/g of DM	
	Raw mulberry cultivars (*Chengxiansang*)	DPPH	147.78 mg of TE/g of DM	
		ABTS	19.62 mg of TE/g of DM	
		FRAP	44.71 mg of TE/g of DM	
	1% grape seed and grape marc meal extract	ABTS	283 μmol/L	[163]
	Grape residue silage	Reducing power	44.6 mg GAE 1^−1^	[140]
Sheep	Control diet	ORAC	197.09 µmol eq. Trolox/g DM	[164]
	5% *Tithonia tubiformis*		201.94 µmol eq. Trolox/g DM	
	5% *Cosmos bipinnatus*		222.79 µmol eq. Trolox/g DM	
	5% *Tagetes lucida*		255.76 µmol eq. Trolox/g DM	
	Control diet	ABTS	50.97 %	[165]
	100 g/day per head of tomato pomace		51.09%	
	100 g/day per head of grape marc		48.01%	
	75 g/day per head of exhausted myrtle berries		52.32%	
	Control diet	ABTS	71.07%	[166]
	150 mg orange peel essential oil/kg concentrate		67.79%	
	300 mg orange peel essential oil/kg concentrate		70.05%	
	450 mg orange peel essential oil/kg concentrate		76.03%	
	Control diet	FRAP	3.18%	
	150 mg orange peel essential oil/kg concentrate		3.42%	
	300 mg orange peel essential oil/kg concentrate		2.54%	
	450 mg orange peel essential oil/kg concentrate		2.99%	
	Control diet	Commercial ELISA kits	14.71 U/mL	[167]
	50 mg cinnamaldehyde, eugenol, and capsicum oleoresin/kg of diet		20.32 U/mL	
	80 mg cinnamaldehyde, eugenol, and capsicum oleoresin/kg of diet		18.17 U/mL	
Goat	Fed sticky corn	DPPH	19.10%	[168]
	Anthocyanin-rich purple corn		21.58%	
	Control diet	FRAP	1.13 mmol/L	[169]
	6% date palm (*Phoenix dactylifera* L.) seed		1.43 mmol/L	
	12% date palm (*Phoenix dactylifera* L.) seed		1.45 mmol/L	
	18% date palm (*Phoenix dactylifera* L.) seed		1.59 mmol/L	
Yak	0 g/kg of astragalus root extract	Commercial ELISA kits	7.23 U/mL	[170]
	20 g/kg of astragalus root extract		7.87 U/mL	
	50 g/kg of astragalus root extract		8.04 U/mL	
	80 g/kg of astragalus root extract		8.20 U/mL	

## 5. Antioxidant Potential of Thermally Treated Milk

A number of studies have reported changes in the antioxidant capacity of milk subjected to thermal treatment [30,128,171]. Various reactions occur between milk compounds during thermal treatment (e.g., denaturation and aggregation of whey proteins and formation of new complexes). It is noteworthy that many studies indicate that β-LG is the most thermally unstable protein and easily undergoes thermal denaturation [26,39,40,41]. Investigations conducted by Liu et al. [26] confirmed this thesis, as it was shown that β-LG-free milk had approximately 50% lower antioxidant activity than skimmed milk. Thermal treatment (100 °C for 2 min) resulted in the loss of antioxidant activity of β-LG due to the blocking of thiol groups. Brodziak et al. [40] showed a statistically significant (*p* ≤ 0.05 and *p* ≤ 0.01) effect of the type of heat treatment on the content of undenatured whey proteins. UHT (ultra-high-temperature) milk was found to contain severalfold lower levels of β-LG, α-LA, and LF than ESL (extended shelf life) and VHT (very-high-temperature) milk. Similarly, Sakkas et al. [39] reported that peroxidase-positive HTST (high-temperature short-time) milk contained >3 g/L of undenatured β-LG. At higher heating temperatures, they recorded a successive decline in the content of undenatured proteins in milk, especially β-LG (90 °C—1132 mg/L; 100 °C—404 mg/L; 130 °C—57 mg/L). Similar findings were reported by Hammershǿj et al. [41]. They achieved a low β-LG denaturation degree of 2–6% in the HTST pasteurization treatment (72 °C/15 s), whereas 30% β-LG denaturation was noted at higher HTLT temperatures (85 °C/30 s).

Processes to which milk proteins with antioxidant potential are subjected affect the total antioxidant capacity of milk. Heat treatment (temperature, time) of raw milk may inhibit or reinforce the formation of antioxidative compounds in the final product. Ertan et al. [171] determined the total antioxidant capacity of pasteurized and UHT milks using the ABTS and Folin–Ciocalteu methods. They showed higher antioxidant capacity of pasteurized milk determined with both methods than that of UHT milk. Additionally, the total antioxidant capacity of milk was found to increase with the increasing milk fat content. Unal [172] also showed the highest antioxidant activity in full-fat UHT milk, which may be associated with higher contents of fat-soluble antioxidants. Therefore, it should be clearly stated that reducing milk fat leads to a reduction in fat-soluble vitamins. Yilmaz-Ersan et al. [30] assessed the antioxidant potential of pasteurized milk (90 °C for 10 min) using three methods (DPPH, ABT, and FRAP). The thermal treatment reduced the DPPH value but increased ABTS and FRAP, compared with raw milk. This may be related to, for example, the reducing properties of the milk, which were strongly affected by heating. Furthermore, the thermal treatment was able to increase its pro-oxidative activity through both loss of natural antioxidants and generation of new oxidizing molecules in the early stages of the Maillard reaction [173]. The increase in the antioxidant activity of sterilized milk can be attributed to the Maillard reaction (i.e., a chemical reaction between carbonyl and amino groups) mainly between lactose and lysine residues in milk proteins [174]. On the other hand, there are reports pointing to no significant differences between the values of antioxidant capacity of raw, pasteurized, and sterilized milks [121,175]. In a research by Şanlidere [175], the values of the antioxidant activity (ABTS) were 4.02, 4.47, and 4.18 mM Trolox/g, respectively. It should be emphasized that these values increased substantially during simulated gastrointestinal digestion (11.13, 12.33, and 11.88 mmol TE/g, respectively). Similarly, Cloetens et al. [121] showed no significant differences between the values of the antioxidant potential of UHT and pasteurized milk. Other studies demonstrated that pasteurization had no effect on the antioxidant capacity of milk, compared with raw milk, whereas sterilization enhanced this parameter [128]. During milk heat treatment at temperatures above 100 °C, the antioxidant capacity increases due to protein unfolding and exposure of thiol groups, potentially acting as hydrogen donors [128,173].

## 6. Antioxidant Potential of Dairy Products

Many studies indicate that the antioxidant potential of dairy products (yogurt, cheese, kefir) is related to the quality of the raw material and primarily the presence and activity of natural bioactive compounds in milk (i.e., amino acids (including tyrosine and cysteine) and vitamins (e.g., A and E)) [12,98]. The bacterial cultures and plant additives used also have a high impact on the value of the antioxidant potential [3,30,176,177,178,179,180,181]. It has been shown that fermented milk products (yogurt and kefir) and cheese have antioxidant properties and are able to scavenge superoxide, hydroxyl, and peroxide radicals and reactive oxygen species [153,182,183,184,185,186,187,188]. Milk fermentation with lactic acid bacteria contributes to the supply of a huge number of bioactive peptides and free amino acids with different biological activities [131,189]. Various investigations indicate that probiotic strains exhibit important antioxidant properties [101]. As reported by Fardet and Rock [12], probiotic yogurts have higher antioxidant activity than conventional dairy products. This is associated with the release of antioxidant peptides by probiotic strains during fermentation, which increase the antioxidant capacity of products and inhibit lipid peroxidation [101,185]. Numerous studies [3,30,190] have shown the importance of the type of probiotic strains used in the production of fermented milk. Fermented products containing *Lactobacillus acidophilus* are characterized by significantly higher antioxidant activity. In a study conducted by Gjorgievski et al. [3], milk with 3.2% fat content was fermented by various microbial cultures, including symbiotic *Lactobacillus delbrueckii* ssp. *bulgaricus* and *Streptococcus thermophilus* and monocultures of *Lactobacillus acidophilus*, *L. casei*, and *Bifidobacterium bifidus*. Compared with the raw material, all microbial cultures increased the antioxidant activity of the fermented product determined with the DPPH method. The highest value was recorded in the case of milk fermented with the probiotic *Lactobacillus acidophilus* strain (54.86%), and the lowest activity was detected in milk fermented with the symbiotic cultures of *Streptococcus thermophilus* and *Lactobacillus delbrueckii* ssp. *bulgaricus* (45.18%). Additionally, the authors noted that the antioxidant activity of fermented milk decreased during storage. Virtanen et al. [185] determined the antioxidant activity (using ABTS tests) of milk fermented with 25 strains of lactic acid bacteria (LAB). They showed strain-specific antioxidant activity. The highest radical scavenging activity was exhibited by *Leuconostoc mesenteroides* ssp. *cremoris* strains (A and B), *Lactobacillus acidophilus* (ATCC 4356), and *Lactobacillus jensenii* (ATCC 25258), which was associated with the protein proteolysis process. Products with high scavenging activity were shown to have higher amounts of peptides in the molecular weight range of 4–20 kDa, while other products were dominated by large polypeptides and compounds below 4 kDa. Moreover, the amount of hydrophobic amino acids in these fermentates was higher. The authors also used combinations of strains in milk fermentation. A significant increase in antioxidant activity was found in the case of combinations containing the *L. acidophilus* strain, with the highest activity noted in the combination of *L. cremoris* B, *L. lactis*, and *L. acidophilus* (TAA 0.86 mmol/L). The Trolox equivalent value was over fivefold lower in the case of the *L. cremoris* B and *L. lactis* combination (TAA 0.16 mmol/L). Milk fermentation is therefore a very good method for the enhancement of the antioxidant activity of products. Concurrently, it is possible to extend the shelf life of dairy products through the inhibition of lipid peroxidation. Skrzypczak et al. [190] conducted studies to identify milk protein solutions (skimmed milk powder, α-LA, caseinoglycomacropeptide) and strains (*L. helveticus* strains B734, 141, T80, and T105; reference strain *L. helveticus* DSMZ 20075) that are desirable for the manufacture of fermented products with the best antioxidant properties. The highest increase in DPPH scavenging activity (% inhibition) was noted for skimmed milk powder solutions fermented by the *L. helveticus* DSMZ 20075 reference strain (85.98%) and *L. helveticus* T80 (81.66%). In the case of α-LA, the strongest free radical scavenging activity (66.67%) was recorded in nonfermented samples [191]. Gamba et al. [127] showed an increase in antioxidant activity during kefir production from cow milk. Depending on the assessment method used, it ranged from 667.4 (ORAC) to 1033.5 μmol Trolox/mL (ABTS). In kefir produced from this milk, the antioxidant activity increased to 1403.5 and 1412.2 μmol Trolox/mL, respectively. Additionally, the authors assessed “soymilk” and showed significantly higher antioxidant activity than cow milk, which was ascribed to the higher content of polyphenols and vitamin E in the soy drink. There was no change in the activity after fermentation of kefir produced from soymilk [127]. Fiorda et al. [191] reported that kefir drinks based on both cow milk and soymilk were characterized by higher antioxidant activity than raw material. In turn, Yilmaz-Ersan et al. [192] used DPPH, ABTS, and FRAP tests to assess the antioxidant activity of kefir produced from goat milk and showed its antioxidant stability at various stages of fermentation (20 h, assessment every 4 h) and during 21 days of storage. However, a decrease in the total phenolic content in the samples was noted during both fermentation and storage. In subsequent studies [30], the authors evaluated the impact of using starter cultures (kefir grains and commercial cultures) on the antioxidant capacity of kefir from cow and sheep milk. The antioxidant capacity of the kefir samples during fermentation and on storage day 21 was assessed using three tests: ABTS, DPPH, and FRAP. It was shown that the type of milk (cow or sheep) and culture used significantly differentiated the antioxidant activity of kefir. Sheep milk and kefir drinks made from this type of milk had higher antioxidant activity than cow milk. As suggested by the authors, this should be associated with differences in the compositions of both types of milk. The authors noted fluctuations in the antioxidant activity of kefir during fermentation, probably due to the inhibition of microbial enzymes present in kefir grains activated in the initial stages of fermentation [177]. During kefir maturation, the ABTS, DPPH, and FRAP values varied. The ABTS values increased significantly until storage day 14. Gupta et al. [187] reported that the ABTS and DPPH values in the case of cheddar cheese produced with the use of *Lactobacillus casei* ssp. casei 300 and *Lactobacillus paracasei* ssp. *paracasei* increased during the first 4 months of maturation and then significantly decreased. Some natural bioactive components of kefir exhibit a relatively slow rate of free radical scavenging, as large peptides and proteins are slowly hydrolyzed and thus have lower antioxidant activity [181]. As reported by Najgebauer-Lejko and Sady [193], yogurt and kefir have the highest antioxidant activity in comparison with other fermented products available on the market. The combination of milk proteins, mainly casein and β-LG, and polyphenols may increase the antioxidant potential of fermented dairy products, which can thus become new functional foods. The presence of probiotic strains, such as *Lactobacillus casei* or *acidophilus*, enhances the antioxidant activity of yogurt as well.

Cheese is regarded as the main source of bioactive peptides due to the high protein content, the variety of proteolytic enzymes, and the degree of proteolysis during cheese ripening [194,195,196]. The antioxidant potential of milk increases during digestion even 2.5 times, which is associated with the release of antioxidant peptides [12]. Oner and Sardag [80] assessed the peptide profile and antioxidant activity of kashar cheese aged for 3 months. After 90 days of ripening, the cheese exhibited significantly higher antioxidant activity and a greater number of peptide peaks. Changes in antioxidant activity occurring during the maturation of cheddar cheese were reported by Gupta et al. [187]. Cheddar cheese was prepared with *Lactobacillus casei* ssp. casei 300 and *Lactobacillus paracasei* ssp. paracasei 22 and without adjunct cultures. The changes in the antioxidant activity were related to the rate of formation of soluble peptides (proteolysis) in all the samples of cheeses up to the fourth month of ripening. Pisanu et al. [77] detected the presence of 187 bioactive peptides in sheep milk cheeses. Seven of these peptides showed strong antioxidant activity and were products of β-CN proteolysis. Garbowska et al. [194] determined changes in the content of bioactive peptides (anserine and L-carnosine) during the maturation of cheese produced with the addition of *Lactobacillus* (*L. casei* 2639, *L. acidophilus* 2499, *L. rhamnosus* 489, and *L. delbrueckii* 49). After a 5-week maturation period, cheese supplemented with *L. acidophilus* 2499 was characterized by the highest content of L-carnosine and anserine (136.11 mg/kg in total) in comparison with other cheese variants. Revilla et al. [11] analyzed the antioxidant capacity of cheese using the ABTS method. A significant (*p* < 0.05) effect of the season of raw milk collection and the duration of the cheese ripening period on the antioxidant capacity was found. The total antioxidant capacity increased until it reached its maximum after 3 months of maturation of winter milk samples and after 1–4 months in the case of summer milk samples. A direct correlation was observed between the cheese maturation time and the TAC value (r = 0.296, *p* < 0.01). The antioxidant activity of the cheese was also significantly correlated with the vitamin A content (r = 0.399). In other studies [195], which included five French farmhouse cheese varieties—Abondance, Tomme de Savoie, Cantalet, Salers, and Rocamadour—the antioxidant activity of cheese was significantly (*p* < 0.05) correlated with fat-soluble antioxidants, including the content of β-carotene and vitamin E. In addition to bacterial cultures, enrichment with raw materials containing compounds with documented antioxidant activity has an impact on the antioxidant value of dairy products. Enrichment of milk and dairy products with natural plant additives increases the antioxidant potential (Table 5). Extensive research has focused on legume seeds, including soybeans, which are added to dairy products to increase their antioxidant potential. Soybeans contain bioactive phytochemicals (e.g., isoflavones, coumestrol, phytate, saponins, lecithin, phytosterols, and vitamin E). With such a composition, soybeans are regarded as a product and raw material with antioxidant properties contributing to the reduction of the risk of heart disease or lowering the levels of cholesterol [196,197,198]. Soybeans are also recognized as a product with high content of protein, fiber, vitamins, and minerals [199]. With the use of the DPPH method, Shori [200] evaluated the antioxidant activity of yogurt from cow and camel milk supplemented with soybeans. The antioxidant activity of the soybean-supplemented yogurt (from both cow and camel milks) was higher than that of the control. Similar findings were reported by Gamba et al. [127], who showed that soymilk had higher antioxidant capacity than cow milk. In another study, Shori et al. [201] assessed the effect of the addition of mangosteen juice to yogurt on its antioxidant potential. The authors used the DPPH method also in this study. The control yogurt stored in refrigeration conditions for 14 days exhibited antioxidant activity in the range of 17–19%, whereas the value of this parameter in the phytomix-3+mangosteen-supplemented yogurt ranged from 60% to 62% in the first week of storage and reached up to 54% in the second week. This study, therefore, proves the possibility of extending the shelf life of yogurt supplemented with phytomix-3+mangosteen, as indicated by the high antioxidant content recorded for 14 days of storage. In addition, mangosteen fruits contain xanthone, which neutralizes free radicals and supports the immune system [202]. Shori and Baba [178] used neem (*Azadirachta indica*) leaf extract as an additive to yogurt. *Azadirachta indica* is used in traditional medicine for the treatment of diabetes and hypertension [203]. It is characterized by high antioxidant potential due to the high content of vitamin C and riboflavin. Higher antioxidant activity (DPPH) was shown by the *Azadirachta indica*-supplemented yogurt in comparison with traditional yogurt (i.e., 30.1 ± 5.1% and 23.5 ± 5.0%, respectively) [179]. During the 28-day storage, the DPPH value increased and reached 53.1 ± 5.0% and 35.9 ± 5.2%, respectively. Dried grape pomace has also been added to yogurt as an alternative source of antioxidant dietary fiber [204]. As shown by the study, dried grape pomace can be used in the production of yogurt not only to increase the content of fiber and total phenolics but also to delay lipid oxidation during refrigerated storage.

Flavoring additives, especially fruit flavors, play an important role in the production of yogurt. Studies by Olas [205] on various types of fruit, including berries, showed that their presence in the diet may constitute an antioxidant barrier against the development of cancer or DNA mutations. Fruit additives are also an excellent prebiotic due to the presence of dietary fiber, which supports the proper function of the gastrointestinal tract. Unal [172] compared the antioxidant activity of various dairy products from the local Turkish market (UHT milk, yogurt, fresh cream cheese, and kefir) using the DPPH test. Fermented milk products containing berries (strawberry, blueberry, blackberry, raspberry) had significantly higher (*p* < 0.05) antioxidant activity than other products. Lee et al. [206] produced fermented milk with the addition of *Cudrania tricuspidata* fruit, which is rich in xanthones and flavonoids. The antioxidant activity of fermented milk determined with different methods (DPPH, ABTS, FRAP) was enhanced by the addition of *Cudrania tricuspidata*. In comparison with the control, the 3% additive concentration contributed to an increase in DPPH (from 1.94 to 3.40 μM TE/mL), ABTS (from 0.31 to 0.64 μM TE/mL), and FRAP (from 0.19 to 1.84 μM TE/mL) radical scavenging activities. It should be noted, however, that consumers evaluating the sensory value of the product accepted only the 0.5% or 1% addition of *Cudrania tricuspidata*. Ni et al. [207] enriched yogurt with extracts from salal berry and black currant pomace. The drink supplemented with black currant pomace had the greatest potential to inhibit the activity of α-glucosidase (>90%), which was probably related to the release of peptides from caseins during the fermentation process. Perna et al. [208] enriched yogurt with chestnut and sulla honeys. Compared with the control, the yogurts with honey were characterized by higher antioxidant activity (ABTS and FRAP). In particular, yogurts with the addition of chestnut honey exhibited higher ABTS and FRAP values, which were closely associated with their high antioxidant activity associated with the highest contents of phenolic acid and flavonoids. Yogurts are also supplemented with herbal additives (e.g., lemon balm, lilac flowers, or seeds such as linseed or chia). In their study, Vuksan et al. [209] reported that the consumption of 25 g of chia seeds with 50 g of glucose reduced postprandial glycemia and appetite in comparison with the control group receiving 25 g of flax with 50 g of glucose or 50 g of glucose alone. As reported by Porter and Bode [210], elderberry exhibits strong antiviral properties. Moreover, WCRF (World Cancer Research Fund International) report and meta-analyses carried out on consumers of milk and fermented milk products showed that this group of products may be a preventive factor against prostate, breast, colon, and stomach cancers [211]. The addition of fruit as a natural source of antioxidants to dairy products extends their shelf life and has an enhancing effect on human health. As indicated by Singh et al. [212], citrus peel, which is regarded as a waste, is a rich source of natural phenolic compounds and carotenoids acting as antioxidants, protecting cells from free radical damage and helping to reduce the risk of many chronic diseases. Due to the presence of antioxidant compounds, citrus peel can be used as a functional additive to food (e.g., dairy products). Ramos et al. [213] assessed the effect of green mate, cloves, lemongrass, and sweet potato pulp on the antioxidant capacity of fermented milk. The addition of a freeze-dried extract (1 g per 100 g of product) containing 87.5% cloves and 12.5% green mate to yogurt significantly (*p* < 0.05) increased the total phenol content (to 54.14 vs. 5.28 mg GAE/100 g in plain yogurt) and FRAP antioxidant capacity (up to 289.96 vs. 31.40 mg AAE/100 g in plain yogurt) in the fermented product. The addition of sweet potato pulp improved the sensory acceptance of the fermented products. Muniandy et al. [214] reported a significant effect of supplementation with green, white, and black tea on the antioxidant activity (FRAP, DPPH, and ferrous ion chelating activity) of yogurts during 21 days of cold storage. The tea-supplemented yogurts exhibited higher antioxidant activity than the plain variant (control) throughout the storage period.

Various investigations indicate that the addition of extracts from red ginseng (*Panax ginseng*) [215] or blackberry flowers (*Rubus ulmifolius*) [216] may increase the antioxidant capacity of yogurts. Fiorda et al. [191] used various functional substrates (hydrolyzed soybean extract, colostrum, and honey) to design new probiotic drinks using kefir grains as a starter culture. Fermentation was carried out at 30 °C for 24 h, and the physicochemical composition and functional aspects were determined. It was found that the honey-based kefir drink exhibited higher antioxidant activity and sensory quality than the traditional kefir drink. Its microbiological composition showed a high level of lactic acid bacteria and yeast (over 106 CFU/mL), mainly the potentially probiotic strains of *Lactobacillus statsumensis*, *Leuconostoc mesenteroides*, *Bacillus megaterium*, and *Lachancea fermentati*. Therefore, honey may be an ideal alternative substrate for the production of a functional culture-based drink, especially for vegans and lactose-intolerant consumers. The antioxidant activity of WPC (whey protein concentrate) and sea algae (spirulina) was assessed as well. A combination of WPC and spirulina increased TAC (58 μmol/g of liver tissue) and lowered the level of cholesterol (58 mg/dL) and malondialdehyde (MDA; a lipid peroxidation marker) [217]. The authors ascribed the activity of WPC to the content of Cys and associated the activity of spirulina with the content of β-carotene, tocopherol, and phycocyanins. Enrichment of cheeses to improve their antioxidant capacity was investigated as well. Da Silva et al. [218] determined the effect of the type of extract (from whole grapes, seeds, and skins) and its concentration (0.1%, 0.2%, and 0.3%) added to cheese milk on the recovery of polyphenols in cheese. As shown by the authors, commercial grape extracts can be used as functional ingredients in the production of cheese without an adverse effect on their yield. The polyphenol recovery rate from whole grape and grape seed extracts was approximately 0.63 at a 0.1% concentration and decreased with the increasing concentration of the extract in milk. Higher rates of polyphenol recovery were observed in the case of the grape seed extracts (0.87) with no concentration effect. The authors emphasize that the consumption of several grams of such cheese can provide the same amount of polyphenols as 1 L of grape juice. Similarly, Marchiani et al. [219] showed an increase in total phenol content and radical scavenging activity (DPPH) in Italian ripened cheeses (Toma and cheddar) after supplementation with dried grape pomace (GP). As highlighted by the authors, it is necessary to add at least 1.6% of GP to achieve a significant increase in the antioxidant activity of cheese. Studies conducted by other authors also showed an increase in the oxidative stability of GP-supplemented cheeses during storage, which is attributed to the antioxidant effect of phenolic compounds contained in the additive. The sensory attractiveness of the cheeses was found to increase as well. Additionally, Marinho et al. [220] compared the antioxidant activity of cheeses that were coated or uncoated with rosemary leaves. They showed that, after 60 days of ripening, the rosemary-coated cheeses were more resistant to the oxidation process than the uncoated cheeses. In subsequent studies, the possibility of using pomegranate peel (*Punica granatum*) extract as a new natural cheese preservative was assessed [221]. Kalari cheese was treated with various concentrations of pomegranate peel extract (0%, 1%, and 2%). A significant (*p* < 0.05) effect of the extract on the oxidative stability of lipids was observed, as the treated products exhibited significantly (*p* < 0.05) lower values of TBARS (mg malondialdehyde/kg) and FFA (% of oleic acid). Similarly, pine needle extract (*Cedrus deodara* Roxb.) can be used as a new preservative in the production of Kalari cheese, as it was found to improve the oxidative stability of cheese significantly [222].

**Table 5:** Enrichment of milk and dairy products with natural plant additives with high antioxidant potential (own work based on: [178,213,215,223,224,225,226,227,228,229,230,231]).

Samples	Additives	Method	Antioxidant Activity	References
Yogurt	Control (without plain *Allium sativum)*	DPPH	26.4 ± 0.7%	[178]
	Plain *Allium sativum* (Garlic)		37.9 ± 0.8%	
Fermented milk	Control (without herbal extract/sweet potato pulp)	FRAP	31.40 ± 1.40 mg AAE/100 g	[213]
	The optimized herbal extract—containing 87.5% clove (*Syzygium aromaticum*) and 12.5% green mate (*Ilex paraguariensis*) (1 g/100 g)		289.96 ± 46.26 mg AAE/100 g	
	The optimized herbal extract (1 g/100 g) and sweet potato pulp (15 g/100 g)		224.95 ± 3.29 mg AAE 100 g	
	Sweet potato pulp (15 g/100 g)		24.51 ± 0.85 mg AAE 100 g	
Milk	Control (without red ginseng extract)	DPPH	11.8 ± 0.00 μg/mL	[223]
	Milk + red ginseng extract (100 μg/mL)		15.1 ± 0.5 μg/mL	
Yogurt	Control (without red ginseng extract)	DPPH	5.8 ± 0.5 μg/mL	
	Yogurt + red ginseng extract (100 μg/mL)		18.7 ± 1.1 μg/mL	
Fresh cheese	Control (without yerba mate)	ABTS	14.59 ± 0.57%	[224]
	1% yerba mate		38.76 ± 2.18%	
	Control (without yerba mate)	DPPH	2.93 ± 0.10%	
	1% yerba mate		67.30 ± 1.35%	
	Control (without yerba mate)	FRAP	0.14 ± 0.01 mg GAE/g	
	1% yerba mate		0.66 ± 0.11 mg GAE/g	
Yogurt	Natural yoghurt (without green tea infusion)	FRAP	1.04 mmol Fe^2+^ EL ^−1^	[225]
	10% green tea infusion		8.98 mmol Fe^2+^ EL ^−1^	
	Control (without *Rosa spinosissima* fruit extract)	FRAP	0.07 ± 0.02 mM Trolox/L	[226]
	0.2% *Rosa spinosissima* fruits extract		2.45 ± 0.02 mM Trolox/L	
	Control (without *Rosa spinosissima* fruit extract)	DPPH	0.86 ± 0.02 mM Trolox/L	
	0.2% *Rosa spinosissima* fruit extract		0.86 ± 0.03 mM Trolox/L	
	Control (without *Rosa spinosissima* fruit extract)	ABTS	3.18 ± 0.07 mM Trolox/L	
	0.2% *Rosa spinosissima* fruit extract		3.33 ± 0.06 mM Trolox/L	
	Control (without Argel leaf extract)	DPPH	32.60 ± 0.20%	[227]
	0.1 g/100 mL Argel leaf extract		47.22 ± 0.02%	
	Control (without aronia juice)	DPPH	59.47 ± 0.31%	[228]
	3% aronia (*A. melanocarpa)* juice		77.87 ± 0.44%	
	Control (without aronia juice)	ABTS	45.96 ± 0.55%	
	3% aronia (*A. melanocarpa)* juice		70.90 ± 0.26%	
	Control (without riceberry rice extract)	FRAP	5.26 ± 0.52 mmol FeSO_4_/100 g	[229]
	0.125% riceberry rice extract		17.42 ± 0.43 mmol FeSO_4_/100 g	
	0.25% riceberry rice extract		25.64 ± 0.96 mmol FeSO_4_/100 g	
	0.5% riceberry rice extract		41.06 ± 2.60 mmol FeSO_4_/100 g	
	Control (without purple basil in water extract)	ABTS	0.67 ± 0.01 mmol TE/kg	[230]
	0.4% purple basil in water extract		1.17 ± 0.01 mmol TE/kg	
	1% purple basil in water extract		1.76 ± 0.01 mmol TE/kg	
	0.4% purple basil in powder form		1.42 ± 0.02 mmol TE/kg	
	1% purple basil in powder form		2.94 ± 0.04 mmol TE/kg	
	Control (without purple basil in water extract)	DPPH	10.66 ± 0.26%	
	0.4% purple basil in water extract		33.16 ± 0.17%	
	1% purple basil in water extract		41.92 ± 0.09%	
	0.4% purple basil in powder form		25.32 ± 0.17%	
	1% purple basil in powder form		43.42 ± 0.17%	
	Control (without red ginseng extract)	DPPH	62.50 ± 4.82%	[215]
	0.5% red ginseng extract		94.46 ± 2.34%	
	1% red ginseng extract		94.85 ± 0.11%	
	1.5% red ginseng extract		94.85 ± 0.07%	
	2% red ginseng extract		94.26 ± 0.31%	
	0 % safflower petal ethanol extract	DPPH	3.24 ± 0.62%	[231]
	1% safflower petal ethanol extract		2.79 ± 0.85%	
	0 % safflower petal hot water extract		5.81 ± 0.61%	
	1% safflower petal hot water extract		10.66 ± 1.21%	

## 7. Conclusions

Summing up, it should be stated that the antioxidant capacity of milk and dairy products is mainly associated with the content of antioxidant components (i.e., proteins), which are rich sources of sulfur amino acids, vitamins A, E, and C, or β-carotene. Biopeptides generated during the fermentation or maturation of cheese also exhibit antioxidant activity. The antioxidant capacity is determined with various methods, mainly ABTS, FRAP, and DPPH assays. Research indicates differences in the total antioxidant capacity of milk between animal species, which result from the differences in the chemical compositions of their milk. Sheep and buffalo milks have the greatest capacity. The content of antioxidant components in milk and the antioxidant potential can be modified through animal nutrition (e.g., supplementation of animal diets with various natural additives). The addition of herbal mixtures or by-products from the fruit and vegetable industry to animals’ rations contributes to the improvement of the nutritional value of milk through an increase in the content of bioactive compounds and antioxidant potential. The antioxidant potential of dairy products is associated not only with the quality of the raw material but also with type of heat treatment, bacterial starter cultures, and natural plant additives used in the processing stage. Fermented products, especially when used as probiotic starter cultures, have the highest antioxidant status. The antioxidant activities of products also increase using plant materials that are rich in phenolic compounds and carotenoids. However, it should be emphasized that the results may be difficult to compare, as different antioxidant activity assays are used. It is difficult to compare the results obtained with different methods, considering, for example, different values and units even within the same method (ABTS and DPPH). Neither method is a reference method, so there is no single clear point of reference. Therefore, it would be worth choosing one most objective method of status assessment and applying it in laboratories all over the world.

## Figures and Tables

**Table 1:** Biologically active peptides with antioxidant properties (own work based on: [51,54,57,58,61,65,66,67,68,69,70,71,72,73,74,75,76,77,78,79,80,81,82,83,84,85,86,87,88,89,90,91,92]).

Protein Precursors	Fragment	Sequence	References
Casein proteins			
Goat milk casein	-	VYPE	[57]
	-	FGGMAH	
	-	FPYCAP	
	-	YVPEPF	
	-	YPPYETY	
Cow milk casein			
CGMP	-	VLPVPQK	[61]
	-	QKAVPYPQRDMPI	
β-casein	1–6	RELEEL	[65]
	7–16	NVPGEIVESL	[66]
	60–68	YPFPGPIN	[67]
	59–63	YGFLP	
	59–68	VYPFPGPIPN	[66]
	84–86	VPP	[68]
	106–123	HKEMPFPKYPVEPFTESQ	[66]
	111–119	FPKYPVEPF	
	114–119	YPVEPF	[67]
	142–154	SWMHQPHQPLPPT	[66]
	166–182	SQSKVLPVPQKAVPYPQ	
	169–176	KVLPVPQK	[69,70]
	170–176	VLPVPQK	
	177–183	AVPYPQR	
	166–175	SQSKVLPVPQ	[71]
	170–175	VLPVPQ	
	176–182	KAVPYPQ	
	183–190	RDMPIQAF	
	178–183	VPYPQR	[72]
	191–193	LLY	[68]
	193–202	YQEPVLGPVR	[73]
	199–209	GPVRGPFPIIV	[74]
	98–105	VKEAMAPK	[58,75,76]
	207–221	QEPVLGPVRGPFPIL	[77,78]
	207–219	QEPVLGPVRGPFP	[78]
	212–219	GPVRGPFP	[51]
	209–220	PVLGPVRGPFPI	[78]
	209–221	PVLGPVRGPFPIL	
	212–220	GPVRGPFPI	[51]
ĸ-casein	24–33	KYIPIQYVLS	[66]
	29–41	QYVLSRYPSYGLN	
	28–30	IQY	[79]
	30–32	YVL	
	51–65	INNQFLPYPYYAKPA	[66]
	66–77	AVRSPAQILQWQ	
	81–95	NTVPAKSCQAQPTTM	
	96–106	ARHPHPHLSFM	
	108–110	IPP	[68]
	115–131	DKTEIPTINTIASGEPT	[66]
αS1-casein	1–9	RPKHPIKHQ	[80]
	7–21	KHQGLPQEVLNENLL	[66]
	26–40	APFPEVFGKEKVNEL	[81,82]
	27–35	PFPEVFGKE	[66]
	39–40	EL	[81]
	80–90	HIQKEDVPSER	[58]
	90–94	RYLGY	[82,83]
	90–96	RYLGYLE	[67]
	91–96	YLGYLE	
	92–94	LGY	
	141–143	EL	[81]
	143–149	AYFYPEL	[75,76,81,84,85]
	143–148	AYFYPE	[67]
	144–149	YFYPEL	
	145–149	FYPEL	
	146–149	YPEL	[81]
	148–149	EL	
	176–192	APSFSDIPNPIGSENSE	[66]
αS2-casein	89–95	YQKFQY	[82]
	89–91	YQK	[85]
	92–95	FPQY	
	130–138	NAVPITPTL	[86]
	171–173	YQK	[85]
	174–181	FALPQYLK	[79]
	202–207	PYVRYL	[76,79]
Cow milk whey proteins			
α-LA	19–29	WYSLAMAASDI	[54]
	50–53	YGLF	[67]
	99–108	VGINYWLAHK	[87]
β-LG	15–20	VAGTWY	[88]
	19–29	WYSLAMAASDI	[54,89]
	42–46	YVEEL	
	58–61	LQKW	[90]
	95–101	LDTDYKK	
	72–79	IAEKTKIP	[87]
	84–91	IDALNEK	[71,90]
	92–100	VLVLDTDYK	
	102–105	YLLF	[67]
	145–149	MHIRL	[54,89]
Proteins from human milk (β-casein)	154–160	WSVPQPK	[91,92]
		

**Table 2:** Selected methods for the determination of antioxidant activity (own work based on: [121,122]).

Method	Principle	Observations
DPPH	In the presence of an antioxidant compound, reduction of the purple-colored stable 2,2-diphenyl-1-picrylhydrazyl (DPPH) radical to yellow 2,2-diphenyl-1-picrylhydrazine	Yellow color of the substance assessed visually or analyzed spectrophotometrically
ABTS	Antioxidants lead to the reduction of the cation radical ABTS^. +^ – 2,2-azinobis-(3-ethylbenzothiazoline-6-sulfonate, causing discoloration of the blue-green solution	Discoloration of the solution assessed visually or analyzed spectrophotometrically
FRAP	Monitoring the antioxidant donor capacity by the measurement of the reduction of the iron(III) complex with 2,4,6-tris(2-pyridyl)-1,3,5-triazine ([Fe^3+^ – (TPTZ)_2_ ]^3+^) to an intense blue complex [Fe^2+^ – (TPTZ)_2_ ]^2+^	Spectrophotometric analysis
ORAC	Antioxidants inhibit free radical-induced oxidation of a fluorescent probe, which shows a decrease in fluorescence during the reaction	Fluorimetric analysis

**Table 3:** Total antioxidant capacity (TAC) of raw milk (own work based on: [30,125,126,127,128,129,130]).

Raw Material	Method				References
	ABTS^•+^	FRAP	DPPH	ORAC	
Cow milk	21.48 ^a^	1.41 ^a^	3.14 ^a^	-	[30]
	-	-	24.3 ^c^	-	[126]
	1033.5 ^b^	-	-	667.4 ^b^	[127]
			18,89 ^c^		[128]
Buffalo milk	7.38 ^e^	38.9 ^d^	31.5 ^c^	-	[129,130]
	-	-	31.8 ^c^	-	[126]
			20.11 ^c^		[128]
Goat milk	6.80 ^e^	23.45–26.71	20.86–23.22	-	[125,130]
	74.36	-	19.53–23.57 ^c^	-	[131]
			18.17 ^c^		[128]
Sheep milk	33.18 ^a^	5.82 ^a^	8.70 ^a^		[30]
	7.78 ^e^		27.28 ^c^		[128,130]
Camel milk	-	-	18.57 ^c^	-	[128]

^a^ Results expressed as milligrams of Trolox equivalents (TE) per 100 mL of the sample. ^b^ Result expressed in μM of Trolox equivalent mg/mL. ^c^ DPPH%. ^d^ Result expressed in µmol/L. ^e^ Result expressed in FeSO_4_ Eq mg/100 g.

## Data Availability

Not applicable.
